# Understanding the critical rate of environmental change for ecosystems, cyanobacteria as an example

**DOI:** 10.1371/journal.pone.0253003

**Published:** 2021-06-18

**Authors:** Bregje van der Bolt, Egbert H. van Nes

**Affiliations:** Department of Environmental Sciences, Wageningen University, Wageningen, The Netherlands; University of Florida, UNITED STATES

## Abstract

Recently it has been show that in some ecosystems fast rates of change of environmental drivers may trigger a critical transition, whereas change of the same magnitude but at slower rates would not. So far, few studies describe this phenomenon of rate-induced tipping, while it is important to understand this phenomenon in the light of the ongoing rapid environmental change. Here, we demonstrate rate-induced tipping in a simple model of cyanobacteria with realistic parameter settings. We explain graphically that there is a range of initial conditions at which a gradual increase in environmental conditions can cause a collapse of the population, but only if the change is fast enough. In addition, we show that a pulse in the environmental conditions can cause a temporary collapse, but that is dependent on both the rate and the duration of the pulse. Furthermore, we study whether the autocorrelation of stochastic environmental conditions can influence the probability of inducing rate-tipping. As both the rate of environmental change, and autocorrelation of the environmental variability are increasing in parts of the climate, the probability for rate-induced tipping to occur is likely to increase. Our results imply that, even though the identification of rate sensitive ecosystems in the real world will be challenging, we should incorporate critical rates of change in our ecosystem assessments and management.

## Introduction

In the recent years the notion that ecosystems can have tipping points has received considerable attention. The term ‘tipping point’ is loosely defined as a threshold point in conditions after which runaway change brings a system to a new state. Under current levels of climate change the likelihood of such transitions in ecological systems is increasing [1,2]. These critical transitions are of great concern because recovery is difficult due to hysteresis. Several mechanisms can lead to critical transitions, but all mechanisms have in common that self-enforcing feedbacks [3] cause the critical transitions. In the mathematical literature three classes of transitions between states are distinguished: bifurcation-induced, noise-induced and rate-induced tipping [4].

**Fig 1:**
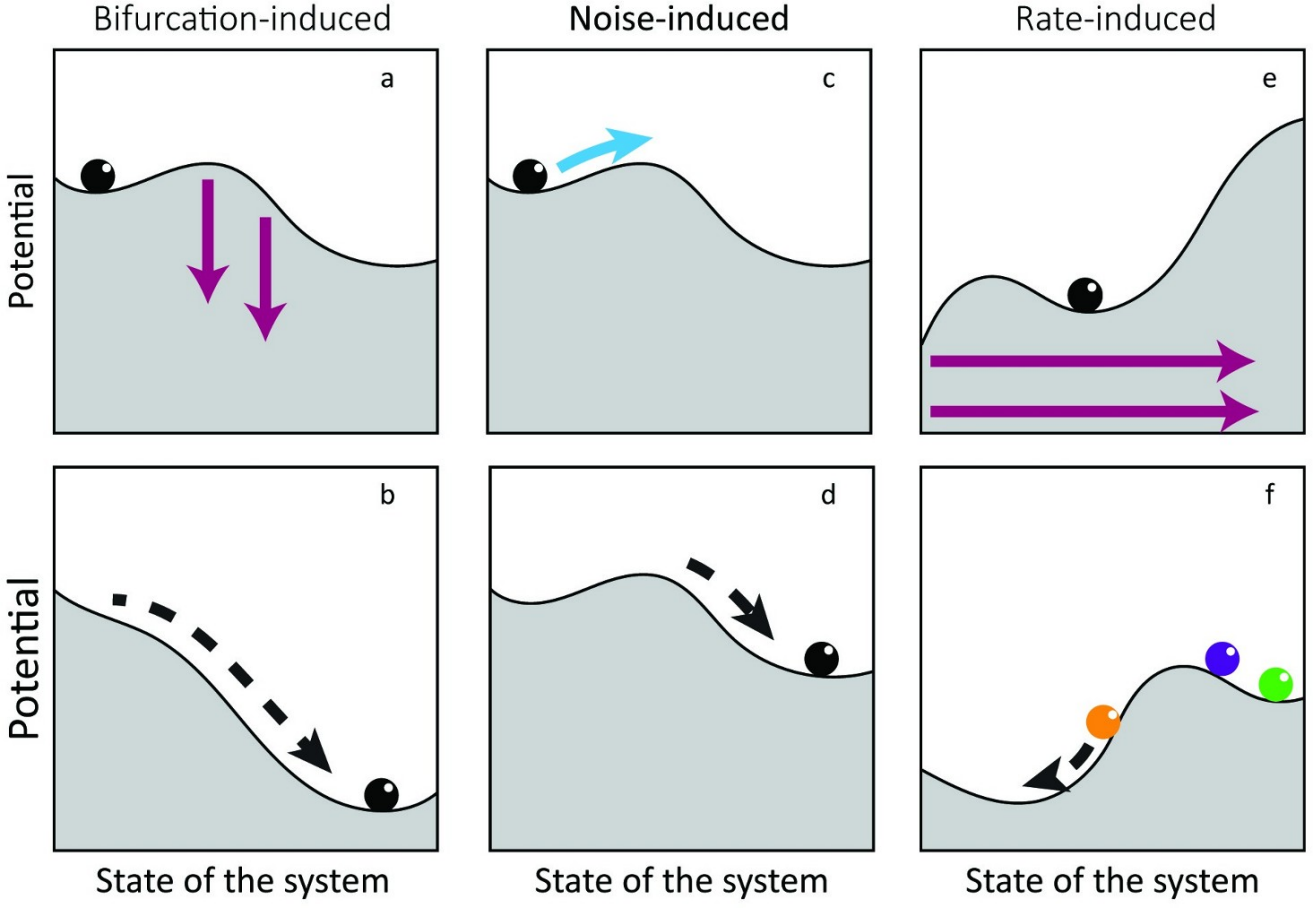
Three types of tipping in a system with alternative stable states. Three types of tipping: Bifurcation-induced (a,b), noise-induced (c,d) and rate-induced (e,f). The upper panels show the initial stability landscapes. Pink arrows show the change in the stability landscape, and the blue arrow shows a change in the state of the system. The lower panels show the stability landscapes at the tipping point, and the dotted arrows indicate the self-enforced change of the system.

Bifurcation-induced tipping is due to a gradual change in external condition (Fig 1A and 1B). As a result of the change in conditions, the resilience of the current state erodes until the system reaches a bifurcation point at which the state becomes unstable and the system shifts to an alternative state [3]. In the case of noise-induced tipping, not a change in external conditions, but a perturbation in the system state, results in a shift from one state to another. When the perturbation is large enough to bring the system across the unstable equilibrium (top of the hill between two valleys, Fig 1C), the system shifts to the other equilibrium [3,5].

Rate induced tipping is caused by a high speed of change in external conditions rather than the absolute level. It has been described recently [4] and can occur only in susceptible systems, i.e., systems that are ‘forward basin unstable’ [6]. This is the case if an instantaneous change in environmental conditions can bring the system to an alternative basin of attraction while no bifurcation point is crossed [6]. Rate-tipping can only be understood if we consider that a system is not always in a steady state and that the equilibrium can change if conditions change. If the external conditions change fast compared to the response rate of the system, the system cannot track the changing equilibrium. As a result, the actual system state can deviate from the stable state. If the change in external changes is below the critical rate, the system state lags behind the changing steady state but stays in the same basin of attraction (Fig 1F, purple ball). If the external changes are above a critical rate, the system lags so far behind the changing equilibrium, that it is brought out of the basin of attraction (Fig 1F, orange ball). Subsequently, the system shifts to another stable state. If the system can respond fast to the changing equilibrium, the system will approximately remain in steady state (Fig 1F, green ball). Rate-induced tipping, can also occur in excitable systems where a fast change in conditions cause the system to cycle once [7]. While there is a vast amount of literature on bifurcation-induced and noise-induced tipping, there are only few studies that describe rate-induced tipping in ecology [8–10].

In light of the ongoing rapid environmental change, it is important to better understand when an ecosystem might be sensitive to rates of change. A candidate system for such rate-induced tipping seems to be a population of cyanobacteria. Cyanobacteria are sometimes toxic keystone taxa that may dominate freshwater and marine ecosystems [11]. Experiments have shown that in chemostats, phytoplankton communities can have tipping points and show critical slowing down before the transition [12,13]. It has been proposed that a self-enforcing feedback that involves photoinhibition can cause these alternative stable states [12,13]. Photoinhibition is a decrease in the rate of photosynthesis as a result of high light. High light levels cause damage to the photosynthetic machinery of the cells, which lowers the photosynthetic rates, or protective mechanisms to avoid damage can lower the rate of photosynthesis [14,15]. This means that although phytoplankton need light for photosynthesis, too much light lowers their productivity. High concentrations of phytoplankton may reduce photoinhibition under high light conditions by self-shading, which implies that there is a self-enforcing feedback [13,16]. As a result of this feedback, phytoplankton communities can maintain a high biomass under light levels that would prohibit growth in a low biomass system. When the light intensity reaches a certain threshold, the shading becomes insufficient to prevent photoinhibition. As a result, biomass decreases, the system becomes even more vulnerable to the high light, and the biomass decreases even further, resulting in a shift to a collapsed state [13]. Due to this positive feedback, the system has alternative stable states for a range of incoming light intensities. Therefore it can show both bifurcation- and noise-induced tipping.

Light is a two-edged sword for the growth of phytoplankton, they need it but too much light can be harmful. Because of this optimum for incoming light intensity, this system might also be sensitive to rates of change of light. Our hypothesis is that if you start from a low biomass and you increase the light intensity gradually, the systems tracks the changing equilibrium and the biomass grows. However, if the light intensity is increased faster, the phytoplankton biomass cannot increase fast enough to provide enough shading and consequently, the system collapses.

Previous work on rate-induced tipping in ecosystems describe the effects of gradual change [9,10] or pulses [8] in environmental conditions, using deterministic models [8–10]. In reality, however, systems are often subject to a regime of stochastic perturbations, called environmental variability. Recent studies show that changes in the temporal autocorrelation of climatic variables may increase the chance of critical transitions in climate-sensitive systems that are close to a bifurcation point [17,18]. This leaves the question how such change in the temporal autocorrelation of climate variability may affect the likelihood of rate-induced transitions. If the rate of environmental change is below, but close to the critical rate, does the temporal autocorrelation of the environmental variability then impact the probability of undergoing a rate-induced transition?.

In this paper we study a model of phytoplankton growth in a chemostat, and show that for realistic parameter settings [12] there can be rate-induced tipping if light levels increase rapidly in this system. We focus on three different types of changes in environmental conditions: a gradual increase to a new level, a pulse perturbation, and a regime of perturbations in combination with a gradual increase to a new level. After we have explored the conditions for which the model shows rate-induced tipping, we discuss the implications of these results for other ecosystems and ecosystem management under current rates of climate change.

## Material and methods

### Model

To explore if phytoplankton communities are sensitive to the rate of change in light intensity, we use a simple model that describes the photoinhibition of phytoplankton in a chemostat [19]. The changes in the phytoplankton concentration of the chemostat can be described as follows:

$$\frac{dA}{dt}=P_{av}\;(I_{z}(z,A))A-lA$$ (1)

Where *A* is the algae concentration (g m^-3^) and *P*_*av*_*(I*_*z*_*(z*,*A))* is the average production rate in the water column (d^-1^) as function of the light availability. The total loss rate *l* (d^-1^) is assumed to be proportional to the phytoplankton concentration implying a constant respiration and flushing rate. In the chemostat the light comes from the side and the light is attenuated in the water due to light absorption and scattering. The light intensity in the water column follows the Lambert-Beer law. According to this formula, the light intensity expressed as photon flux *I*_*z*_ (μmol m^-2^ s^-1^) decays exponentially with position *z* (m):

$$I_{z}(z,A(t))=I_{in}e^{-K_{d}\;z-k\;A(t)z}$$ (2)

Where *Iin* is the light intensity at the top of the water column, *K*_*d*_ is the light attenuation coefficient of particles and substances in the water (m^-1^). This light attenuation depends on particles and substances in the water. Therefore, also the phytoplankton biomass has an effect on the light attenuation coefficient. This effect is approximately linear with biomass [20], so in our model *Iz* is a function of both *z* and *A*. *k* is the specific extinction of phytoplankton (m^2^ g^-1^). The spatial averaged light limitation of phytoplankton in the chemostat can be calculated by integration over the total water column *D* (m):

$$P_{av}(I_{z}(z,A(t))=\frac{1}{D}\int_{0}^{D}P_{z}(I_{z}(z,A(t)))dz$$ (3)

The relationship between light availability *I*_*z*_ and the relative phytoplankton production *P*_*z*_ can be described with the Peeters-Eilers equation, that includes the effect of photoinhibition [21]:

$$P_{z}(I_{z}(z,A(t)))=P_{max}\frac{I_{z}(z,A(t))}{a\;I_{z}{\left(z,A(t)\right)}^{2}+bI_{z}(z,A(t))+c}$$ (4)

Where *P*_*max*_ is the maximum growth rate of phytoplankton (d^-1^). The precise meaning of the parameters *a*, *b*, and *c* are hard to interpret. Therefore, Peeters and Eilers introduced two new parameters: *I*_*opt*_, the optimum light availability for production, and *I*_*k*_, that defines the slope of the curve at *I*_*z*_ = 0. Specifically, *I*_*k*_ is the light availability where the maximum production is reached if the initial slope was continued. The parameters *a*, *b*, and *c* can be expressed as function of these two new parameters:

$$a=\frac{I_{k}}{I_{opt}{}^{2}},b=1-2\frac{I_{k}}{I_{opt}},\text{and}\;c=I_{k}$$ (5)

The integral of the phytoplankton production (Eq 4) can be solved analytically [21]. The default values of the parameters of the model (Eqs 3 and 4) describe phytoplankton in a chemostat [12] and can be found in Table 1. The model was analyzed using Grind for MATLAB (http://www.sparcs-center.org/grind.html) which uses a Runge-Kutta numerical solver (ode45). For continuation of bifurcations, GRIND uses MatCont version 6.10 [22].

**Table 1:** Description of the parameters and state variables with their initial conditions and default values.

Variable	Value	Description	Units
*A*	6.1779	Phytoplankton biomass	g m^-3^
*I*_*in*_	100	Light intensity	μmol m^-2^ s^-1^
*D*	0.05	Size of the water column	m
*I*_*k*_	40	Slope of the light curve at I_z_ = 0	μmol m^-2^ s^-1^
*I*_*opt*_	150	Optimum light intensity of phytoplankton	μmol m^-2^ s^-1^
*k*	3	Specific light attenuation coefficient of phytoplankton	m^2^ g^-1^
*k*_*d*_	10^−5^	Background light attenuation coefficient	m^-1^
*l*	0.4	Loss of phytoplankton due to flushing	day^-1^
*P*_*max*_	0.49	Maximum productivity	day^-1^

### Simulated incoming light intensities

We focus on three different kinds of change in the incoming light intensity: a gradual increase to a new level, a pulse perturbation, and a stochastic regime of perturbations. First, we increased the light intensity with different rates to check when the model can track the equilibrium. After that, we analysed how pulses in the light intensity affect the phytoplankton community in the chemostat. Lastly, we determined how the system is affected by a stochastic regime of perturbations. The analysis of phytoplankton dynamics under constant environmental conditions can be found in the S1 File.

In the first scenario, we increased the light intensity from a low value (*I*_*in*_ = 100 μmol m^-2^ s^-1^) to a high value (*I*_*in*_ = 800 μmol m^-2^ s^-1^) with a constant rate. This scenario is called ‘steady drift’ [4] and is implemented in the model as follows:

$$\frac{dI_{in}}{dt}=\{\begin{array}{l} r\\ 0 \end{array}\;\begin{array}{r} if\;I_{in}<I_{in,\max}\\ otherwise \end{array}$$ (6)

Where *r* is the rate by which the light intensity is increased. The initial conditions are the equilibrium states (A*) for the low light conditions (*I*_*in*_ = 100: A* = 6.1779).

Next, we explored the potential effects of a pulse in a rate-sensitive system. We exposed the model to a brief pulse in the value of the parameter *I*_*in*_. The effect of such a pulse is different than a gradual increase in light intensity, because not only the relative rate, but also the duration of the pulse determines whether the system tips or not. Therefore, we varied both the rate and the duration of pulse. During the pulse we let *I*_*in*_ approach a maximum value exponentially (this is called ‘unsteady drift’ [4], *I*_*in*_ will remain at the maximum value for a specific duration, after which *I*_*in*_ decreases exponentially back to the start value. We ran the model for different values of *r*, ranging from 0.1 to 0.4 with a step of 0.001. The duration of the pulse was defined by *t*_*end*_, which varied from 115 to 180 with a step of 0. The formula for the pulse in the light intensity is:

$$\frac{dI_{in}}{dt}=\{\begin{array}{l} r(I_{in,max}-I_{in})\\ r(I_{in,min}-I_{in}) \end{array}\;\begin{array}{r} if\;t>t_{start}\;and\;t<t_{end}\\ otherwise \end{array}$$ (7)

Where *I*_*in*,*max*_ is the maximum value of the pulse of 800 μmol m^-2^ s^-^1, and *I*_*in*,*min*_ the base light intensity.

The simulations started with the ecosystem in equilibrium with low light *I*_*in*,*min*_ and we applied the pulse after 100 time steps. In this model, a pulse will always have a temporary effect, as the system is not bistable at the initial conditions, implying that it can recover even from very low values. Reverting the change will therefore eventually result in recovery. This recovery, however, will take very long compared to the systems’ timescale.

In a deterministic situation, only the starting conditions and the rate of change affect whether the system shifts or not, but in a stochastic situation, this is no longer true. We tested how the autocorrelation of hypothetical environmental variables affects the probability of invoking a critical transition in a changing environment. We created time series of hypothetical environmental fluctuations *T*_*a*_ using the following stochastic differential equation:

$$\frac{dT_{a}}{dt}=-\gamma T_{a}+\beta \frac{dW}{dt}$$ (8)

Where *β* is a parameter scaling the variance of the noise, *dW/dt* is a Wiener process where the increments are normally distributed. *γ* is ≥ 0 and corresponds to negative feedbacks that act to restore any anomaly to the mean, *T*_*a*_ = 0. The smaller *γ*, the larger the autocorrelation of *T*_*a*_; *γ* = 0 corresponds to an unbounded random walk (Brownian motion). We solved the equation using an Euler-Maruyama scheme with time step 1. With this scheme and time step, the relation between *γ* and the autocorrelation *α* of the discrete time series generated with this model can be described as α(1) = 1-γ (sampling time step = 1).

In reality, when the temporal autocorrelation of the environmental variability increases, the variance of the variability also changes (see S7 Fig in S1 File). The variance of a process can increase as a response to weakening feedbacks, but there are also mechanisms that reduce the variance while increasing the persistence. For example, when the heat capacity or inertia of a system changes [18,23,24]. To gain insight into how increased temporal autocorrelation in the environmental variability might affect rate-induced tipping, we study the effect of time-correlation in isolation. Therefore, we scaled the parameter *β* in such a way to keep the expected variance of *T*_*a*_ at a constant value using the following equation:

$$\beta =\sigma \sqrt{\frac{\left(1-\alpha^{2}\right)}{\Delta t}}$$ (9)

We ran the model with time series of *T*_*a*_ with different levels of autocorrelation *α* (*γ* of Eq 8 1 = (1-α)/dt), ranging from 0 to 0.9 with an increment of 0.05, and different levels of standard deviation (*σ*), ranging from 1 to 8 with an increment of 0.25. We increased the incoming light intensity from 100 to 800 (Eq 6), with a rate r that is just below the critical rate (r_crit_ = 50.0104, r = 48), to test whether the type of environmental variability in combination with the gradual change can result in rate-induced tipping. For each level of autocorrelation and standard deviation *σ* we repeated the simulations 1,000 times to determine what percentage of the runs collapsed.

Environmental variability, or noise, can be added to the model as additive, or multiplicative noise. For the model and results of this model with additive noise, see S1 File. Here, we focus on the effect of environmental variability when it affects the incoming light intensity (multiplicative noise). This means that the stochastic fluctuations are added to the incoming light intensity in the following way:

$$\frac{dI_{in}}{dt}=\{\begin{array}{c} r+T_{a}(t)\\ T_{a}(t) \end{array}\;\begin{array}{c} if\;I_{in}<I_{in,max}\\ otherwise \end{array}\frac{dI_{in}}{dt}=r(I_{in,max}-I_{in})+T_{a}(t)$$ (10)

Where *r* is the rate by which the light intensity is increased (r_crit_ = 50.0104, r = 48) and *I*_*in*.*max*_ the maximum value of the incoming light intensity of 800 μmol m^-2^ s^-^1. We did the same analysis in a model with unsteady drift; details and the results can be found in the S1 File.

## Results

### Gradual increase of light intensity

**Fig 2:**
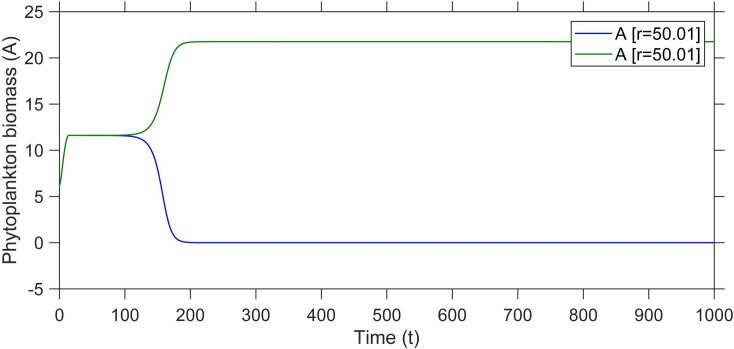
Simulated effects of the rate of change (r) in light intensity (I_in_) on phytoplankton biomass. In this model the light intensity is changed gradually from 100 to 800 with two different rates (r = 50.104, and r = 50.105).

We started all simulations from a state in which the phytoplankton population size was adapted to the low light conditions, which implies that the biomass is relatively low. If the light intensity is increased slowly, the phytoplankton could grow fast enough for self-shading. When we increased the light intensity very rapidly, the system shifted to the other state because the growth-rate of the phytoplankton was too slow to keep up with the increased light intensity (blue line, Fig 2).

**Fig 3:**
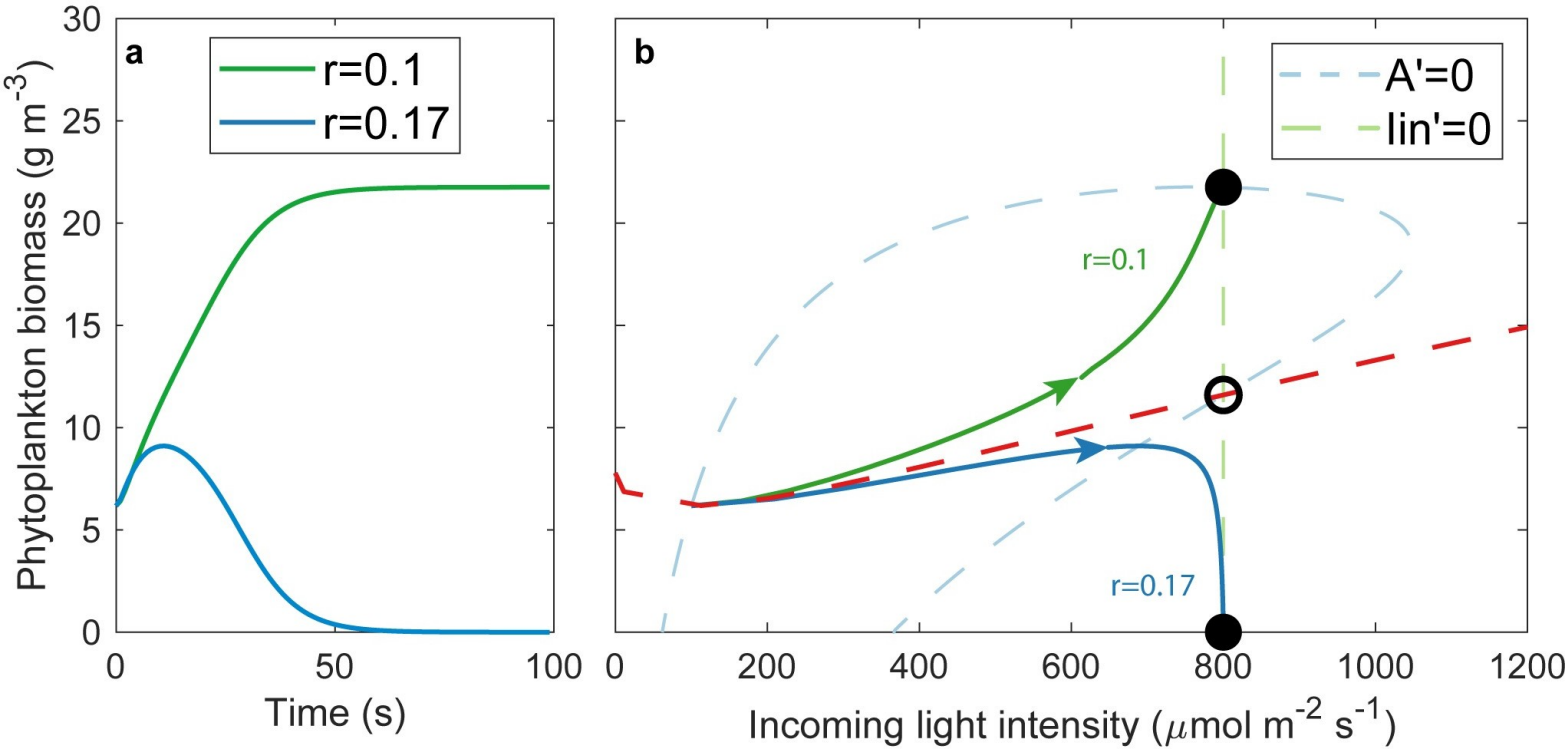
Simulated effects of the rate of change (r) in light intensity (I_in_) on phytoplankton biomass. a) If the light intensity is increased slowly, the phytoplankton can grow fast enough to provide self-shading and the system will move towards a high-biomass equilibrium (green line). If the rate is too fast, the phytoplankton cannot grow fast enough to provide shade and the system collapses (blue line). b) The (I_in_, A)-phase plane of the model. The blue dotted line is the nullcline of the phytoplankton biomass. The green dotted line is the nullcline for I_in_, which is a vertical line at I_in,max_ (I_in,max_ = 800 for these settings). If I_in_ changes very fast, the system is brought past the separatrix (red dotted line) and moves to the collapsed state (blue line), while if I_in_ changes more gradually, the system can follow the equilibrium and the phytoplankton biomass increases (green line).

**Fig 4:**
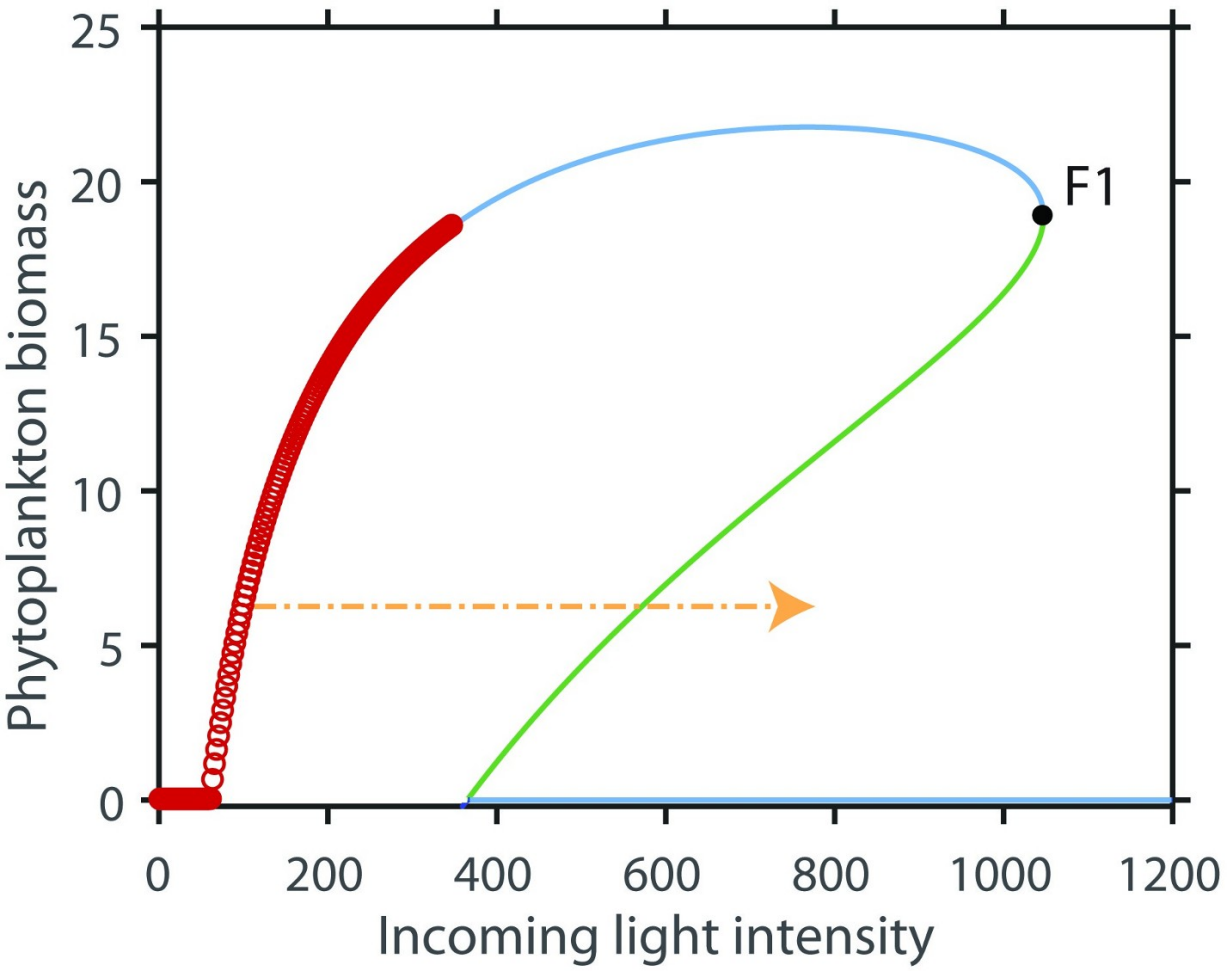
Initial conditions that are forward basin instable. The red dots indicate the initial conditions that are forward basin stable. The blue line indicates the stable equilibrium, the green line the unstable equilibrium and F1 is the fold bifurcation. When a change in the incoming light intensity occurs with infinite speed (horizontal line, as for example the orange arrow) and crosses the unstable equilibrium, the new equilibrium is in another basin of attraction than the initial equilibrium, while the fold bifurcation F1 is not passed. If this is the case, the system is forward basin instable.

The reason why this model has rate-induced tipping can also be understood from the (*I*_*in*_, *A*)-phase plane of the model with unsteady drift (Fig 3B, see S1 File for details). If *I*_*in*_ changes gradually, the system can follow the equilibrium and the phytoplankton biomass increases (green line, Fig 3B). If *I*_*in*_ changes too fast, however, the system is brought past the unstable equilibrium and as a consequence, the phytoplankton biomass collapses (blue line, Fig 3B). The critical rate for r-tipping depends on the initial conditions and the maximum light intensity. The conditions where rate tipping is potentially possible have “forward basin instability” [6]. This is the case if an instantaneous change in environmental conditions can bring the system to an alternative basin of attraction while no bifurcation point is crossed [6]. In a simple model with one state variable, this instability can be read from the phase plane (Fig 4), A change with infinite speed from an initial condition to a higher value for the incoming light intensity can be visualized as a horizontal line (like the orange arrow). If that straight line crosses the unstable equilibrium (Fig 4, green line), the system is forward basin instable, because the new equilibrium is in another basin of attraction than the initial equilibrium and the fold bifurcation F1 is not passed. Initial conditions for which rate-tipping could occur in this model, are indicated in Fig 4 (red dots). In this model, for rate-tipping to be possible, the maximum light intensity needs to be large enough that an instantaneous change to the maximum light intensity, can bring the system past the unstable equilibrium (see Fig 4). If we allow the system to start outside the equilibrium this means that for higher initial biomass conditions, the maximum light intensity needs to be larger than for low initial biomass conditions for rate-tipping to be possible.

### Effect of light intensity pulses

In the previous section we have explored the impact of a gradual increase of incoming light on tipping, showing that when the increase in light intensity goes too fast, the system cannot track the changing equilibrium. Next to gradual increase, drivers of environmental change can also increase for a brief period of time. For example, when environmental conditions are reversed as a result of an implemented policy. Furthermore, the increasing frequency and duration of extreme weather events [25] like heat waves and droughts also act as pulses on ecosystems.

**Fig 5:**
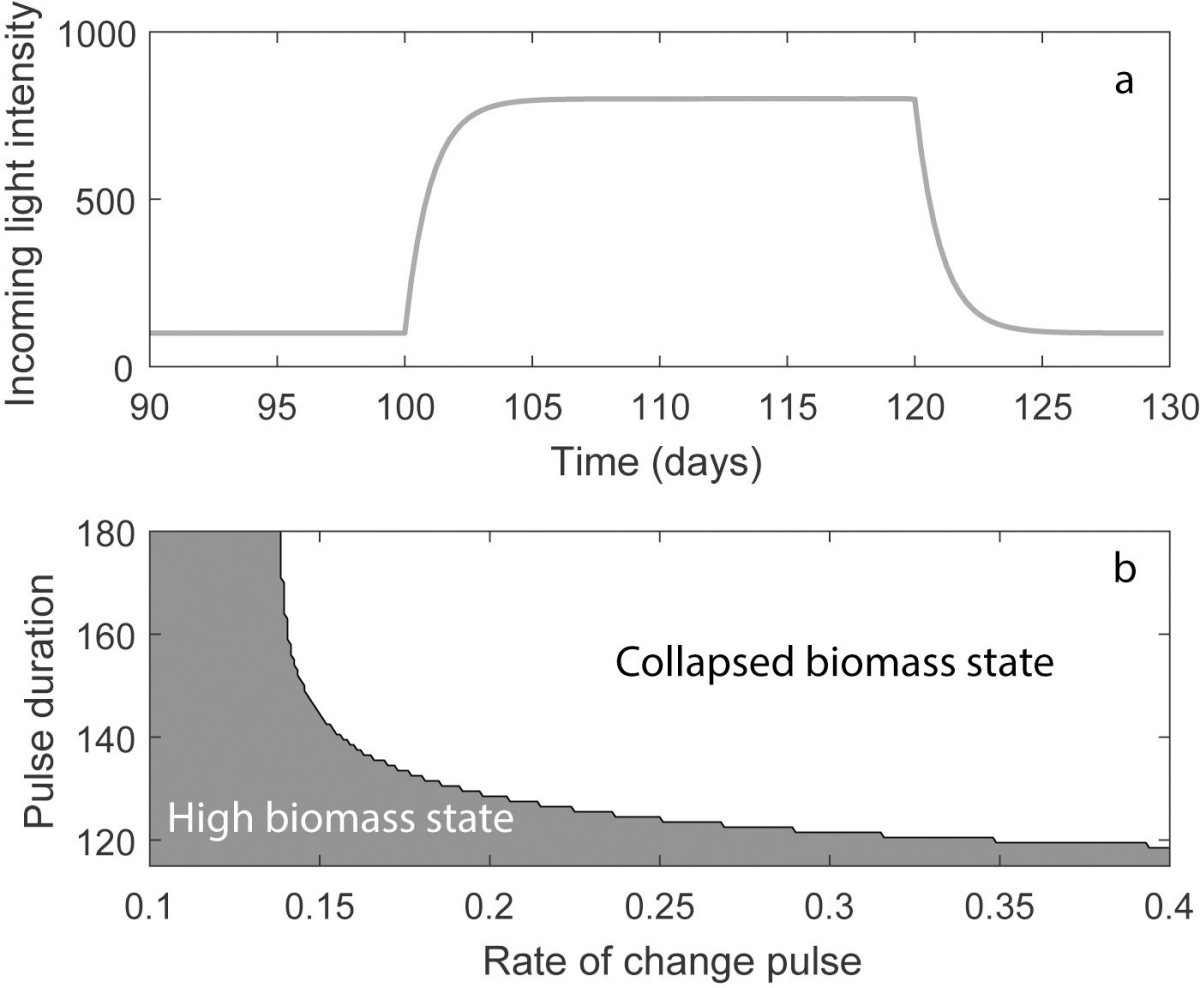
Simulation experiment altering pulse duration and intensity for an exponential increase of the light intensity. (a) Example time series of I_in_ with a pulse of 140 days. (b) The response of the phytoplankton biomass to pulses that increase and decrease exponentially with different rates of change (x-axis) and different durations of the pulse (y-axis). The high biomass state refers to the state in which the phytoplankton population is able to maintain the biomass, and in the collapsed biomass state self-shading was not sufficient to prevent photoinhibition and as a result, the biomass collapsed. The rate of change pulse (parameter r) is the rate by which the incoming light intensity is changed during the pulse.

As with the gradual increase in light intensity, if the rate of change is too fast, the system cannot track the changing equilibrium and the system is brought in the alternative basin of attraction and the phytoplankton starts collapsing. In contrast to gradual increase, the pulse should last long enough to cause a collapse. If the light intensity is lowered already before the phytoplankton biomass is collapsed, the biomass returns to the initial biomass value. For each specific rate there is a specific minimum duration that is needed for the system to collapse. For higher rates, shorter durations are needed for the biomass to collapse (Fig 5B).

With our initial conditions, the critical rate for rate-tipping to occur is 0.1378 day^-1^. With this rate, the duration of the pulse needs to be at least 382 days for the system to collapse (i.e. biomass < 10^−8^ g m^-3^). If the rate is very high (r>1000), the critical duration of the pulse is still around 112 days, indicating that for a pulse to have an effect on the phytoplankton community, the duration of the pulse should be longer than 112–382 days, depending on the rate of change.

### Effect of stochastic perturbation regimes in light intensity

In reality, ecosystems are subject to a regime of perturbations in the environmental conditions, this is also called environmental variability. In different environments, the variability can have different power spectra. Previous research indicates that systems with bifurcation-induced tipping are sensitive to the frequencies of the environmental variability. Longer periods of adverse conditions result in an higher probability of tipping in a more time-correlated environments [17]. Because our previous analyses show that the phytoplankton biomass is sensitive to the duration of pulses, increased time-correlation in the environmental variability might also affect the probability of a collapse in biomass.

**Fig 6:**
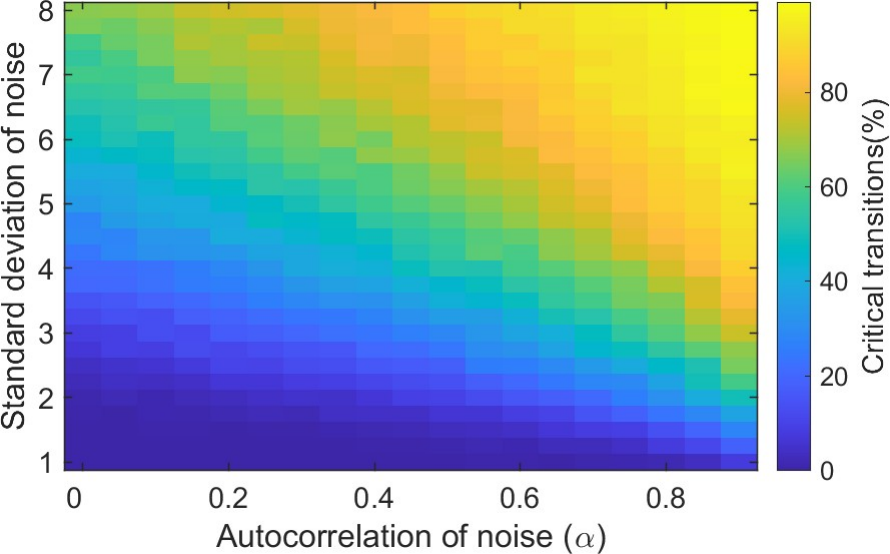
The response of the phytoplankton biomass to an increase in the light intensity with stochastic fluctuations on the light intensity. Combined effects of autocorrelation and the standard deviation of the fluctuations on the percentage of runs in which the phytoplankton biomass collapses when the rate of change is 48.

Our results (see Fig 6) indicate a higher probability of shifting in a system with more time-correlated stochasticity. When the environmental variability affects the incoming light intensity, the fluctuations are in the driver. When the fluctuations are uncorrelated, the fluctuations are very rapid but the phytoplankton biomass does not have time to respond to the short changes. When the fluctuations are correlated, however, the fluctuations are slower and can cause an temporary increase in light intensity on top of the gradual increase, that results in a collapse of the biomass (see Fig 6).

## Discussion and conclusions

We have shown with this model with realistic parameter settings [12] that speed of change can be critical for the effect that environmental conditions have on phytoplankton. Although our model of phytoplankton is not new, rate tipping has not been described before for this system. Because the parameterization of the model is partly based on the experimental settings and experimental data of Faassen et al. (2015), our results imply that that rate-tipping could occur in an experimental setup, if you increase the incoming light intensity fast enough. This indicates that a phytoplankton community in a chemostat might be a good experimental setting to test under which conditions rate-tipping might occur in a real ecosystem. In addition, we show that a pulse in the conditions can also cause a temporary collapse, and that the kind of stochastic regime can influence the probability of shifting.

With the current rates of environmental change [1,26], the question arise what kind of systems are sensitive to rate-induced tipping. A way to see if a model potentially can have rate tipping is to test for forward basin instability [6]. This can be tested numerically by comparing the effects of a gradual change of the driver to a specific value, with an instantaneous change to the same value of the driver. In our model, there is a range of initial conditions where rate tipping can occur. As our model has one state variable, forward basin instability can easily been seen from the bifurcation diagram. Due to the hump-shaped bifurcation diagram, there are initial conditions where an instantaneous change in conditions (here an increase in light) brings the state to the other basin of attraction (see orange arrow in Fig 3). When the conditions stay long enough at that level the system will tip to the alternative basin of attraction (rate tipping). For models with more than one state variables the conditions of forward basin stability are less obvious, and need to be determined numerically [27]. In addition, rate-tipping can also occur in systems that do not have alternative states, but that have coupled slow and fast non-linear processes [9]. For example, in the climate-carbon cycle model of Luke and Cox (2011), where the soil carbon decomposition rate increase exponentially with soil temperature. In this model, a runaway feedback can arise when the heat from microbial respiration is generated more rapidly than it can escape from the soil to the atmosphere [28]. Another example is the Rosenzweig-MacArthur predator-prey model used by Vanselow, Wieczorek and Freudel (20119) and Siteur et al (2016).

Although it is not difficult to show mathematically if a model can potentially have rate tipping, it is still difficult to predict more intuitively if a certain ecosystem has a critical rate. We know already that for alternative stable states we need to have a positive feedback (Van Nes et al 2016). In addition, systems that are sensitive for rate tipping often include a key process with an optimum. For instance in the model of Scheffer et al. (2009 this optimum was a functional response with an optimum prey density for feeding. The current model includes an optimum light availability for phytoplankton growth. Such optimum conditions are rather common in ecosystems. For instance in the reaction of species growth to temperature, there is often an optimum temperature involved, and species niches are commonly defined as optima (for instance [29]). Although this gives us clues where to look for, the identification of rate sensitive species or ecosystems in the real world might be more difficult; rate-induced tipping has not yet been observed in real ecosystems. It seems hard to prove that real ecosystem tip due to the rate of change only instead of crossing a critical value. A number of experimental studies, however, do show rate-dependent outcomes [30–33]. For example, Perron, Gonzakez and Buckling (2008) [33], showed that both the rate of change of antibiotics concentrations and immigration had a significant effect on the probability of evolving resistance in an experimental population of *Pseudomonas euruginosa*. Whereas Lindsey et al. (2013) [30] allowed hundreds of populations of *Escherichia coli* to evolve under different rates of increase in antibiotics. Their results show that there are more chances for mutations to occur under lower rates of change. One of the factors that makes it difficult to predict the effect of rapid environmental change on ecosystems, is that different species may respond differently to changes in the environment. Besides shifting in abundance, species may respond by shifting their spatial or temporal range, by phenotypic plasticity or by adaptive microevolution [34–37]. Each of these mechanisms may have their own critical rate, but the rate will be species and event specific. Thus, further exploration of rate-sensitivity in ecosystems will require a better understanding of how different species within the ecosystem respond to environmental change. In addition, we need to explore whether ecosystem models that include multiple species with different responses to environmental change, also show rate-induced critical transitions, and if so, whether these systems have similar features. Despite our limited understanding of their occurrence, the possibility of wide-spread rate-induced tipping in ecosystems has profound implications for the way we assess the impact of global change on ecosystems, and the way we manage our ecosystems. Besides identifying and quantifying planetary boundaries in order to create a safe operating space for our ecosystems [38,39], we now also need to incorporate critical rates of change.

## Supporting information

S1 File Supplementary information. This document contains a more detailed description of the used methods and additional analyses. Specifically, this document contains additional information on (1) basic model dynamics (2) unsteady drift (3), unsteady drift and environmental variability, (4) pulse in the incoming light intensity, (5) the effect of scaled environmental variability, and (6) additional figures.
(PDF)

## References

[1] Klein Goldewijk K, Beusen A, Van Drecht G, De Vos M. The HYDE 3.1 spatially explicit database of human-induced global land-use change over the past 12,000 years. Glob Ecol Biogeogr. 2011;20: 73–86. doi: 10.1111/j.1466-8238.2010.00587.x

[2] Bestelmeyer BT, Ellison AM, Fraser WR, Gorman KB, Holbrook SJ, Laney CM, et al. Analysis of abrupt transitions in ecological systems. Ecosphere. 2011;2: art129. doi: 10.1890/ES11-00216.1

[3] van Nes EH, Arani BMS, Staal A, van der Bolt B, Flores BM, Bathiany S, et al. What Do You Mean, “Tipping Point”? Trends Ecol Evol. 2016;31: 902–904. doi: 10.1016/j.tree.2016.09.011 27793466

[4] Ashwin P, Wieczorek S, Vitolo R, Cox P. Tipping points in open systems: bifurcation, noise-induced and rate-dependent examples in the climate system. Philos Trans R Soc London A Math Phys Eng Sci. 2012;370: 1166–1184. doi: 10.1098/rsta.2011.0306 22291228

[5] Dakos V, Carpenter SR, van Nes EH, Scheffer M. Resilience indicators: prospects and limitations for early warnings of regime shifts. Philos Trans R Soc London B Biol Sci. 2015;370: 20130263. 10.1098/rstb.2013.0263.

[6] Ashwin P, Perryman C, Wieczorek S. Parameter shifts for nonautonomous systems in low dimension: bifurcation- and rate-induced tipping. Nonlinearity. 2017;30: 2185–2210. doi: 10.1088/1361-6544/aa675b

[7] Wieczorek S, Ashwin P, Luke CM, Cox PM. Excitability in ramped systems: the compost-bomb instability. Proceedings of the Royal Society of London A: Mathematical, Physical and Engineering Sciences. The Royal Society; 2011. pp. 1243–1269.

[8] Scheffer M, Van Nes EH, Holmgren M, Hughes T. Pulse-driven loss of top-down control: the critical-rate hypothesis. Ecosystems. 2008;11: 226–237.

[9] Siteur K, Eppinga MB, Doelman A, Siero E, Rietkerk M. Ecosystems off track: rate-induced critical transitions in ecological models. Oikos. 2016;125: 1689–1699. doi: 10.1111/oik.03112

[10] Vanselow A, Wieczorek S, Feudel U. When very slow is too fast—collapse of a predator-prey system. J Theor Biol. 2019;479: 64–72. doi: 10.1016/j.jtbi.2019.07.008 31302207

[11] Huisman J, Codd GA, Paerl HW, Ibelings BW, Verspagen JMH, Visser PM. Cyanobacterial blooms. Nature Reviews Microbiology. Nature Publishing Group; 2018. pp. 471–483. doi: 10.1038/s41579-018-0040-1 29946124

[12] Faassen EJ, Veraart AJ, Van Nes EH, Dakos V, Lürling M, Scheffer M. Hysteresis in an experimental phytoplankton population. Oikos. 2015.

[13] Veraart AJ, Faassen EJ, Dakos V, van Nes EH, Lürling M, Scheffer M. Recovery rates reflect distance to a tipping point in a living system. Nature. 2012;481: 357–359. doi: 10.1038/nature10723 22198671

[14] Long SP, Humphries S, Falkowski PG. Photoinhibition of photosynthesis in nature. Annu Rev Plant Physial Plant Mol BioI. 1994;45: 633–62. Available: https://www.annualreviews.org/doi/pdf/10.1146/annurev.pp.45.060194.003221.

[15] Takahashi S, Murata N. How do environmental stresses accelerate photoinhibition? Trends Plant Sci. 2008;13: 178–182. doi: 10.1016/j.tplants.2008.01.005 18328775

[16] Holmgren M, Scheffer M, Huston MA. The interplay of facilitation and competition in plant communities. Ecology. 1997;78: 1966–1975. doi: 10.1890/0012-9658(1997)078[1966:TIOFAC]2.0.CO;2

[17] van der Bolt B, van Nes EH, Bathiany S, Vollebregt ME, Scheffer M. Climate reddening increases the chance of critical transitions. Nat Clim Chang. 2018;8: 478–484. doi: 10.1038/s41558-018-0160-7

[18] Boulton CA, Lenton TM. Slowing down of North Pacific climate variability and its implications for abrupt ecosystem change. Proc Natl Acad Sci. 2015;112: 11496–11501. doi: 10.1073/pnas.1501781112 26324900 PMC4577159

[19] Gerla DJ, Mooij WM, Huisman J. Photoinhibition and the assembly of light-limited phytoplankton communities. Oikos. 2011;120: 359–368. doi: 10.1111/j.1600-0706.2010.18573.x

[20] Megard RO, Settles JC, Boyer HA, Combs WS. Light, Secchi disks, and trophic states1. Limnol Oceanogr. 1980;25: 373–377. doi: 10.4319/lo.1980.25.2.0373

[21] Peeters JCH, Eilers P. The relationship between light intensity and photosynthesis—A simple mathematical model. Hydrobiol Bull. 1978;12: 134–136. doi: 10.1007/BF02260714

[22] Dhooge A, Govaerts W, Kuznetsov YA. MATCONT: a MATLAB package for numerical bifurcation analysis of ODEs. ACM Trans Math Softw. 2003;29: 141–164.

[23] Bathiany S, Van Der Bolt B, Williamson MS, Lenton TM, Scheffer M, Van Nes EH, et al. Statistical indicators of Arctic sea-ice stability-prospects and limitations. Cryosphere. 2016;10. doi: 10.5194/tc-10-1631-2016

[24] Wagner TJW, Eisenman I. False alarms: How early warning signals falsely predict abrupt sea ice loss. Geophys Res Lett. 2015;42: 10,333–10,341. doi: 10.1002/2015GL066297

[25] Coumou D, Rahmstorf S. A decade of weather extremes. Nat Clim Chang. 2012;2: 491. doi: 10.1038/nclimate1452

[26] Joos F, Spahni R. Rates of change in natural and anthropogenic radiative forcing over the past 20,000 years. Proc Natl Acad Sci U S A. 2008;105: 1425–30. doi: 10.1073/pnas.0707386105 18252830 PMC2234160

[27] O’Keeffe PE, Wieczorek S. Tipping phenomena and points of no return in ecosystems: beyond classical bifurcations. arXiv Prepr arXiv190201796. 2019.

[28] Luke C., Cox PM. Soil carbon and climate change: from the Jenkinson effect to the compost-bomb instability. Eur J Soil Sci. 2011;62: 5–12. doi: 10.1111/j.1365-2389.2010.01312.x

[29] Liautaud K, van Nes EH, Barbier M, Scheffer M, Loreau M. Superorganisms or loose collections of species? A unifying theory of community patterns along environmental gradients. Coulson T, editor. Ecol Lett. 2019;22: ele.13289. doi: 10.1111/ele.13289 31134748 PMC6642053

[30] Lindsey HA, Gallie J, Taylor S, Kerr B. Evolutionary rescue from extinction is contingent on a lower rate of environmental change. Nature. 2013;494: 463–467. doi: 10.1038/nature11879 23395960

[31] Bell G, Gonzalez A. Evolutionary rescue can prevent extinction following environmental change. Ecol Lett. 2009;12: 942–948. doi: 10.1111/j.1461-0248.2009.01350.x 19659574

[32] Collins S, de Meaux J. Adaptation to different rates of environmental change in Chlamydomonas. Evolution (N Y). 2009;63: 2952–2965. doi: 10.1111/j.1558-5646.2009.00770.x 19619223

[33] Perron GG, Gonzalez A, Buckling A. The rate of environmental change drives adaptation to an antibiotic sink. J Evol Biol. 2008;21: 1724–1731. doi: 10.1111/j.1420-9101.2008.01596.x 18681913

[34] Holt RD. The microevolutionary consequences of climate change. Trends Ecol Evol. 1990;5: 311–315. doi: 10.1016/0169-5347(90)90088-U 21232381

[35] Charmantier A, McCleery RH, Cole LR, Perrins C, Kruuk LEB, Sheldon BC. Adaptive Phenotypic Plasticity in Response to Climate Change in a Wild Bird Population. Science (80-). 2008;320: 800–803. doi: 10.1126/SCIENCE.1157174 18467590

[36] Chen I-C, Hill JK, Ohlemüller R, Roy DB, Thomas CD. Rapid range shifts of species associated with high levels of climate warming. Science. 2011;333: 1024–6. doi: 10.1126/science.1206432 21852500

[37] Visser ME. Keeping up with a warming world; assessing the rate of adaptation to climate change. Proceedings Biol Sci. 2008;275: 649–59. doi: 10.1098/rspb.2007.0997 18211875 PMC2409451

[38] Scheffer M, Barrett S, Carpenter SR, Folke C, Green AJ, Holmgren M, et al. Creating a safe operating space for iconic ecosystems. Science (80-). 2015;347. doi: 10.1126/science.aaa3769 25792318

[39] Rockström J, Steffen W, Noone K, Persson Å, Chapin FS, Lambin EF, et al. A safe operating space for humanity. Nature. 2009;461: 472–475. doi: 10.1038/461472a 19779433

