# Supplementary material for: Understanding the critical rate of environmental change for ecosystems, cyanobacteria as an example
Source: PLoS One. 2021 Jun 18;16(6):e0253003. doi: 10.1371/journal.pone.0253003 (PMC8213170; doi:10.1371/journal.pone.0253003)
Supplement: S1 File — This document contains a more detailed description of the used methods and additional analyses. Specifically, this document contains additional information on (1) basic model dynamics (2) unsteady drift (3), unsteady drift and environmental variability, (4) pulse in the incoming light intensity, (5) the effect of scaled environmental variability, and (6) additional figures. (PDF) [file pone.0253003.s001.pdf]

Supplementary Information

Understanding the critical rate of environmental change for ecosystems, cyanobacteria as an example

Bregje van der Bolt and Egbert H. van Nes

Department of Environmental Sciences, Aquatic Ecology and Water Quality Management Group,

Wageningen University, The Netherlands

S1. Basic model dynamics

Basic phytoplankton dynamics

The incoming light intensity (I_{in}) affects the growth of phytoplankton, and for specific values of incoming light intensity, the system can have alternative stable states. Under such conditions, the per-capita productivity of phytoplankton first increases with plant biomass due to increased shading and then declines again as a result of competition for light (Fig. S1a). It is easy to see from this figure that phytoplankton biomass has only a positive growth rate at intermediate densities (between E2 and E3 in Fig. 3a); at lower densities there is photoinhibition and at higher densities light limitation. The low biomass state (E2) is an unstable equilibrium, meaning that a small perturbation in the phytoplankton biomass will bring the system either to the high biomass equilibrium (E3), or to extinction (E1, Fig. S1b).

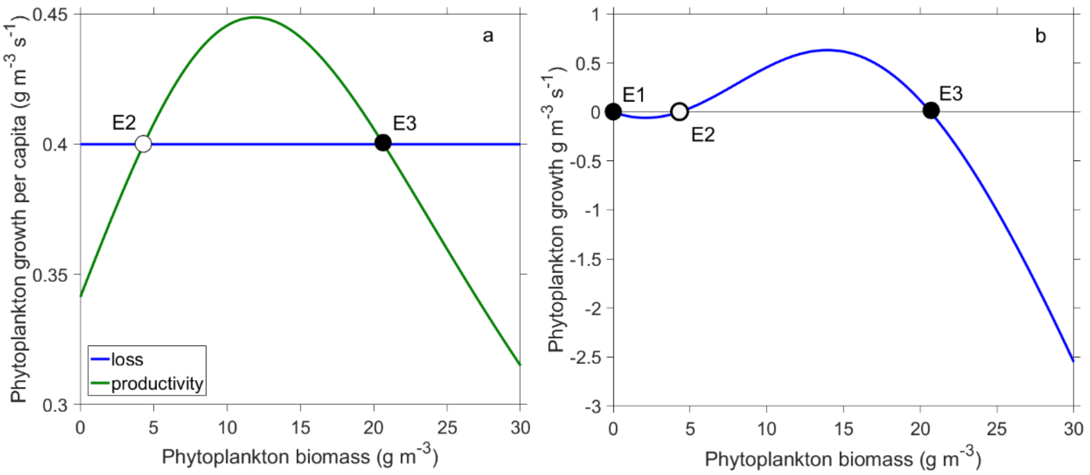

Figure S1. (a) The per capita rate of change in phytoplankton biomass depends on the difference between productivity and losses. At intermediate phytoplanktion biomass the net growth is positive. At lower biomass the the phytoplankton cannot shade each other out, while at higher biomass the competition for light becomes too high. (b) The biomass growth rate as a function of biomass. At the biomass levels where the net growth is zero, the system is in equilibrium. The heavy dots (E1 and E3) are stable equilibria, while the light dot (E2) is an unstable equilibrium. Parameter settings for this figure can be found in Table 1.

If you change the parameter values of the model the rates of change and consequently, the equilibria change. For instance, when you decrease I_{in}, the productivity is always larger than the losses at low biomass, so the collapsed state becomes unstable and there is only one stable equilibrium (solid

dot, Figure S2a). On the other hand, if I_{in} is increased, P1 and P2 collide, implying that for higher I_{in}, the loss rate exceeds the production rate irrespective of the population density (Figure S2c). In this case the only equilibrium left is the collapsed state (*A*=0).

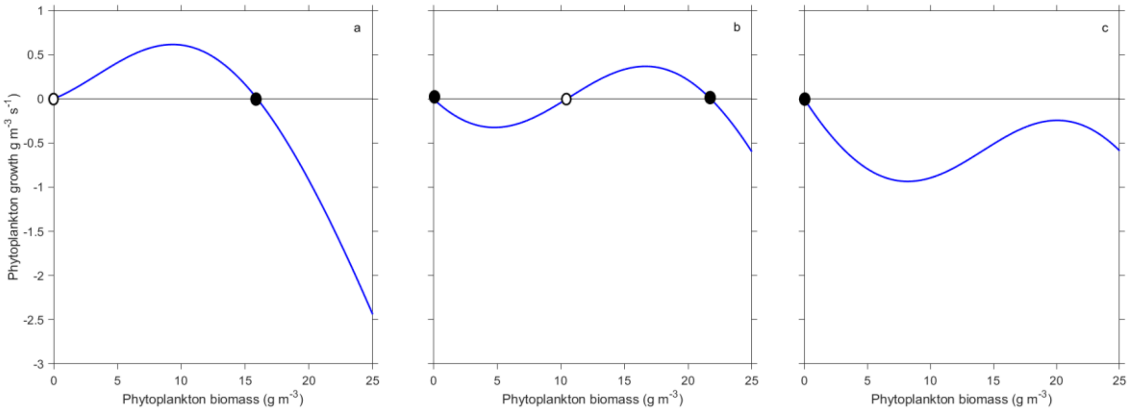

Figure S2. The relation between changes in phytoplankton growth and biomass for different levels of incoming light intensity (I_{in} = 250, 750 and 1250). At the point where the change in phytoplankton biomass is zero, the system is in equilibrium. A black dot indicates a stable equilibrium, an open dot an unstable equilibrium.

Effects of environmental conditions

To explore how the incoming light intensity affects the equilibria of the model, we did a bifurcation analysis with I_{in} ranging from 0 to 1500 μmol m^{-2} s^{-1}. For the default parameter settings (see Table 1), the model has three biological relevant bifurcations within this range of I_{in} (Fig. S3): two transcritical bifurcations and one fold bifurcation. A transcritical bifurcation is a point at which two equilibria cross and exchange stability, and a fold bifurcation is the point at which the stable and the unstable equilibrium merge. In this model, there is a transcritical bifurcation at low light (T1, at I_{in} = 61.6604) below which there is too little light for phytoplankton to grow, and a transcritical bifurcation at intermediate light (T2, at I_{in} = 364.902), above which the light intensity is too high for phytoplankton to colonize an empty system. The fold bifurcation occurs at high light (F1, at I_{in} = 1047.27) and high biomass, so there is high self-shading by phytoplankton (Fig. S3).

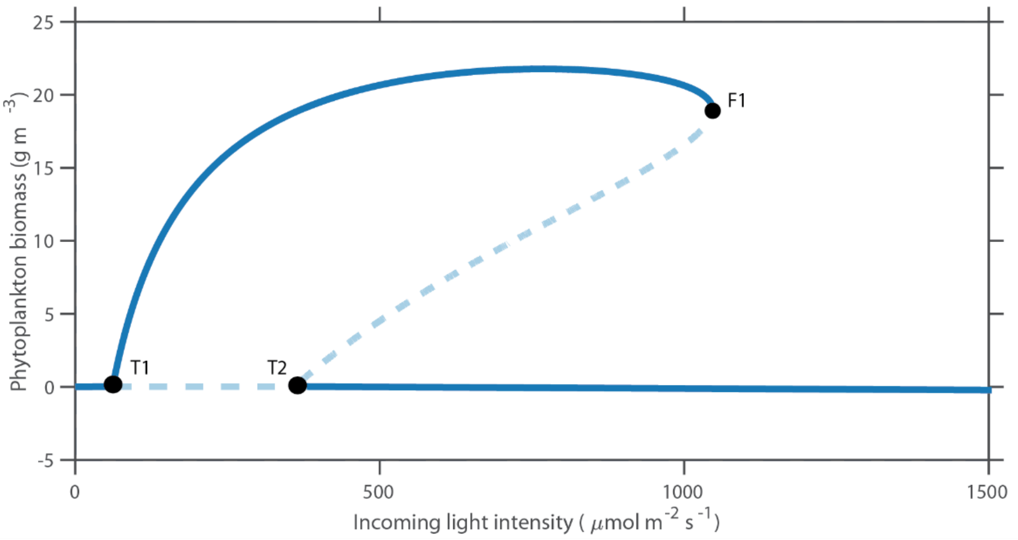

Figure S3. Bifurcations of the phytoplankton for different levels of light intensity (I_{in}) and phytoplankton biomass (A). There are three biological relevant (A >= 0) bifurcations: at T2 there is a transcritical birfucation at low light, below which there is too little light for phytoplankton to grow. At F1 there is a fold bifurcation with high light and high self-shading by the phytoplankton, and at T1 there is another transcritical bifurcation, above this intermediate light intensity, there is no colonization of the collapsed state.

To visualize how the incoming light intensity affects the outcome of the model, we construct a 2 dimensional bifurcation diagram with I_{in} and l, the loss of phytoplankton due to flushing, on the axes. In the diagram we can distinct three regions with different model predictions: no phytoplankton, only phytoplankton and alternative stable states (Fig. S4). The specific flushing rate at which the transcritical bifurcation (blue line,T1 & T2) occurs is dependent on I_{in}. Likewise, the curve that represents the fold bifurcation (green line, F1 & F2), also shows the dependence of the fold bifurcation on l and I_{in}. If I_{in} becomes bigger than I_{opt} (default I_{opt}=150), there exists a window of l for which the systems has alternative stable states (Fig. S4). Of I_{in} is lower than I_{opt}, there is also a region with alternative stable states, but this region only exists for negative phytoplankton biomasses and is therefore not biological relevant (F2 part of the green line, Fig. S4). If l becomes bigger than the maximum productivity (p_{max} = 0.49), positive growth is never possible and phytoplankton cannot exist.

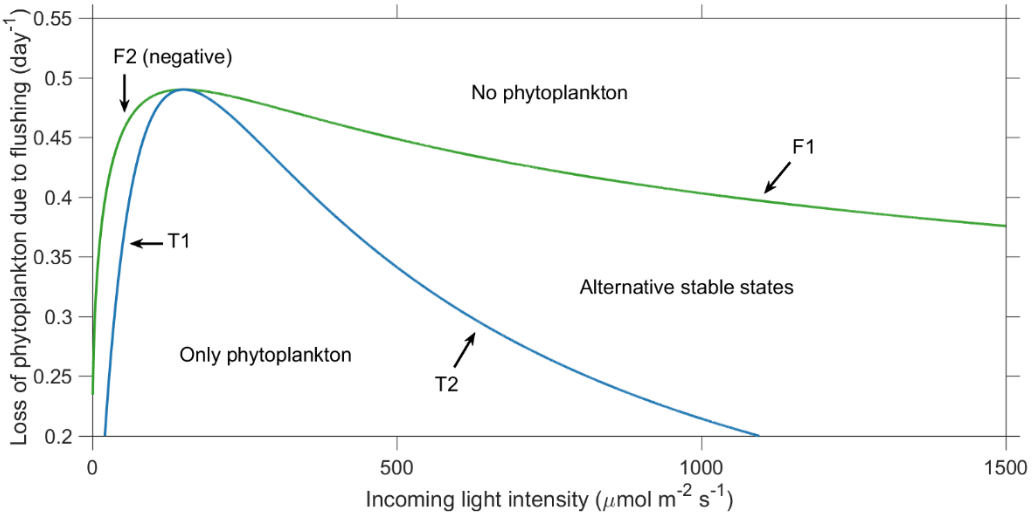

Figure S4. Two-dimensional bifurcation diagram for incoming light intensities between 0 and 1500 μmol m^{-2} s^{-1} and loss of phytoplankton due to flashing between 0.2 and 0.55 per day. The green line shows the fold bifurcations, and the blue line the transcritical bifurcations. The part of the curve that represents the second fold bifurcation (F2) is biologically not relevant, as values for the phytoplankton are negative for this bifurcation. The bifurcations divide the diagram in three distinct regions: parameter combinations in which phytoplankton cannot exist, where there is only phytoplankton and a region in which alternative stable states can occur.

S2. Unsteady drift

Although steady drift is the simplest assumption for the change of the light conditions, there are also other options, like letting the light intensity exponentially approach a maximum value (called ‘unsteady drift’ (Ashwin et al., 2012)). An advantage is that this approach allows for a phase plane analysis in the (I_{in}, A) plane. The unsteady drift is implemented with the following equation:

$$\frac{dI_{in}}{dt} = r\,(I_{in,max} - I_{in}) \tag{S1}$$

With r = 0.1. In Figure S5 one can see the conditions for which rate-tipping is possible. The blue, solid line is the separatrix and above this line, the speed is either too slow to induce rate-induced tipping, or rate-induced tipping is never possible.

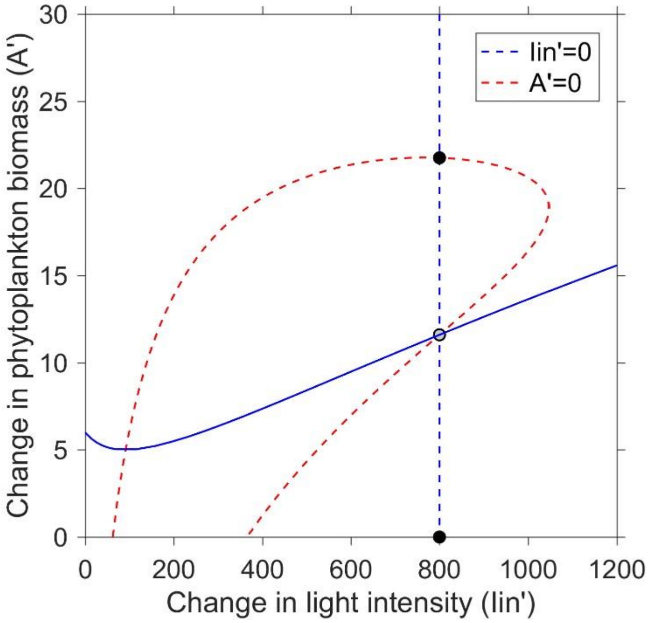

Figure S5. Phase plane analysis at the (I_{in}, A) plane. The blue, solid line is the separatrix, above which rate-induced tipping does not occur.

When the value for *r* is changed, the separatrix changes. With higher values for *r*, tipping becomes easier as the system diverges more from the initial state equilibrium and it becomes more difficult to follow the equilibrium state. For lower rates, the system is more likely to follow the equilibrium and rate-induced tipping does not occur. When *r* becomes larger than 1, the separatrix approaches the saddle almost in a horizontal line (Fig. S6).

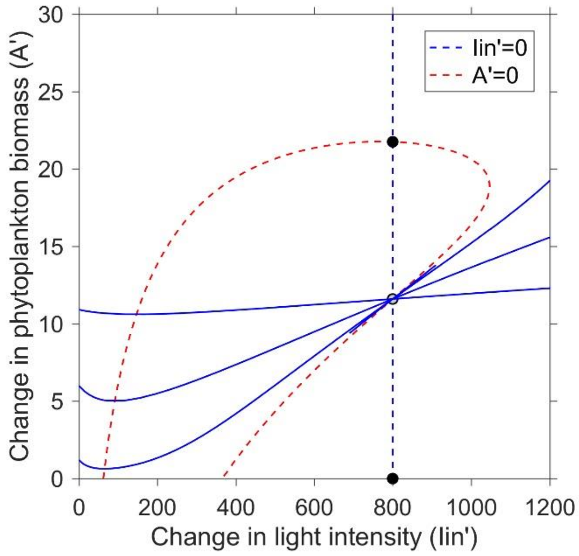

Figure S6. The separatrix in the phase plane analysis for different values of r. The higher the value for r, the larger the range of conditions for which there is rate-induced tipping.

S3. Unsteady drift and environmental variability

In addition to the scenario in which the incoming light increases linearly, and the environmental variability is added to the incoming light intensity (see Main text), we ran two additional scenarios:

1. The incoming light intensity changes exponentially (unsteady drift) and environmental variability affects the system state directly (additive noise)
2. The incoming light intensity changes exponentially (unsteady drift) and environmental variability affects the incoming light intensity (multiplicative noise)

In these scenario's we assume that the environmental variable $T_a(t)$ can have different effects on the phytoplankton biomass. For the first scenario (1), we assumed that the stochastic fluctuations directly act on the phytoplankton biomass in the following way (additive noise):

$$\frac{dA}{dt} = \left(P_{av}\left(I_z(z, A(t))\right) A(t) - l\, A(t) \right) + T_a(t) \qquad \text{(S2)}$$

Where *r* is the rate by which the light intensity is increased (r_{crit} = 0.1378, r = 0.13) and $I_{in.max}$ the maximum value of the incoming light intensity of 800 µmol m^{-2} s^{-}1.

In the second scenario (2), the stochastic fluctuations act on the incoming light intensity in the following way:

$$\frac{dI_{in}}{dt} = r\left(I_{in,max} - I_{in}\right) + T_a(t) \qquad \text{(S3)}$$

Where *r* is the rate by which the light intensity is increased (r_{crit} = 0.1378, r = 0.13) and $I_{in.max}$ the maximum value of the incoming light intensity of 800 µmol m^{-2} s^{-}1.

Similar to the analysis in the main text, we ran the model with generated time series of T_a with different levels of autocorrelation α (γ of Eq.9 1=(1-α)/dt), ranging from 0 to 0.9 with an increment of 0.05, and different levels of standard deviation (σ), ranging from 10 to 80 with an increment of 2.5.

Our results (see Fig. S7) indicate a higher probability of shifting in a system with more time-correlated stochasticity. The stochastic fluctuations in the phytoplankton biomass have an influence on whether the phytoplankton can provide enough shading for the biomass to withstand the incoming light intensity. If the phytoplankton biomass is lower, the critical rate is lower, meaning that the change in

incoming light intensity should be more gradual in order for the phytoplankton to be able to grow enough to provide shading. In an uncorrelated environmental signal (α=0, see Fig. S10a) the fluctuations in the biomass are very rapid but do not last long enough to cause a collapse. In a more time-correlated environment (for example, α=0.8, see Fig. S10c) , however, the fluctuations in the phytoplankton biomass have a longer duration and as a consequence, the biomass can collapse at lower rates.

The results for the second scenario, where the environmental variability affects the incoming light unsteady and the incoming light intensity is increased exponentially, are similar to the runs in which the incoming light intensity is increased linearly (see Fig. S8).

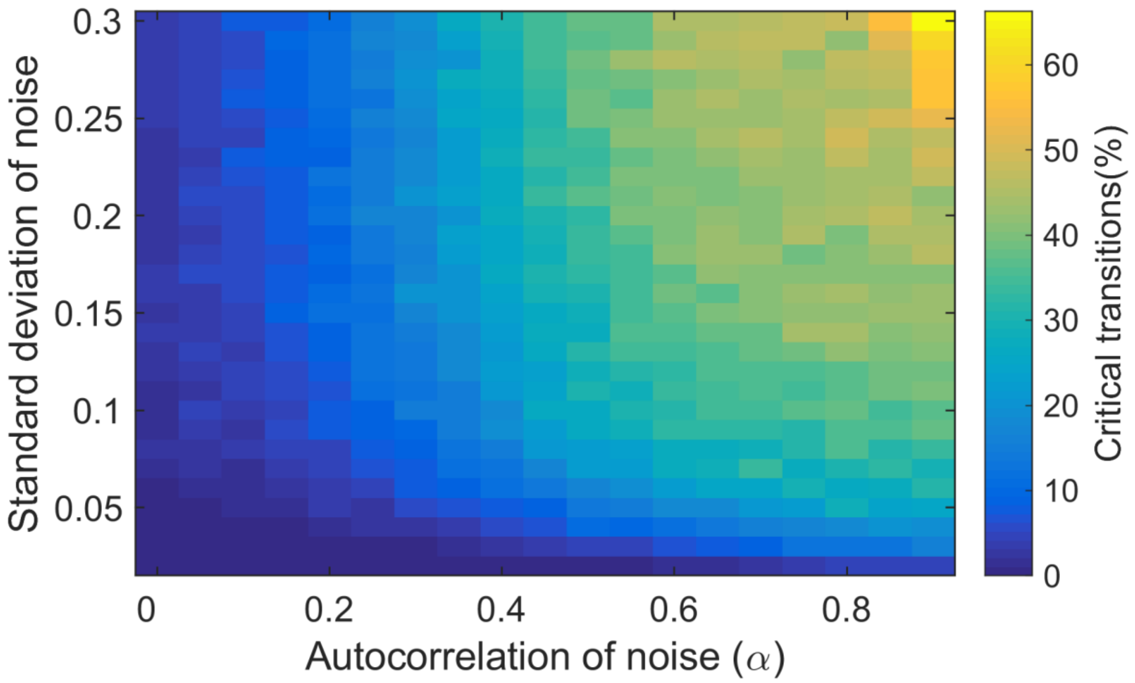

Figure S7. The response of the phytoplankton biomass to an increase in the light intensity in an stochastic environment in the model with unsteady drift (scenario 2). Combined effects of autocorrelation and the standard deviation of the fluctuations in the phytoplankton biomass on the percentage of runs in which the phytoplankton biomass collapses when the rate of change of the incoming light intensity is 0.13 day^{-1}.

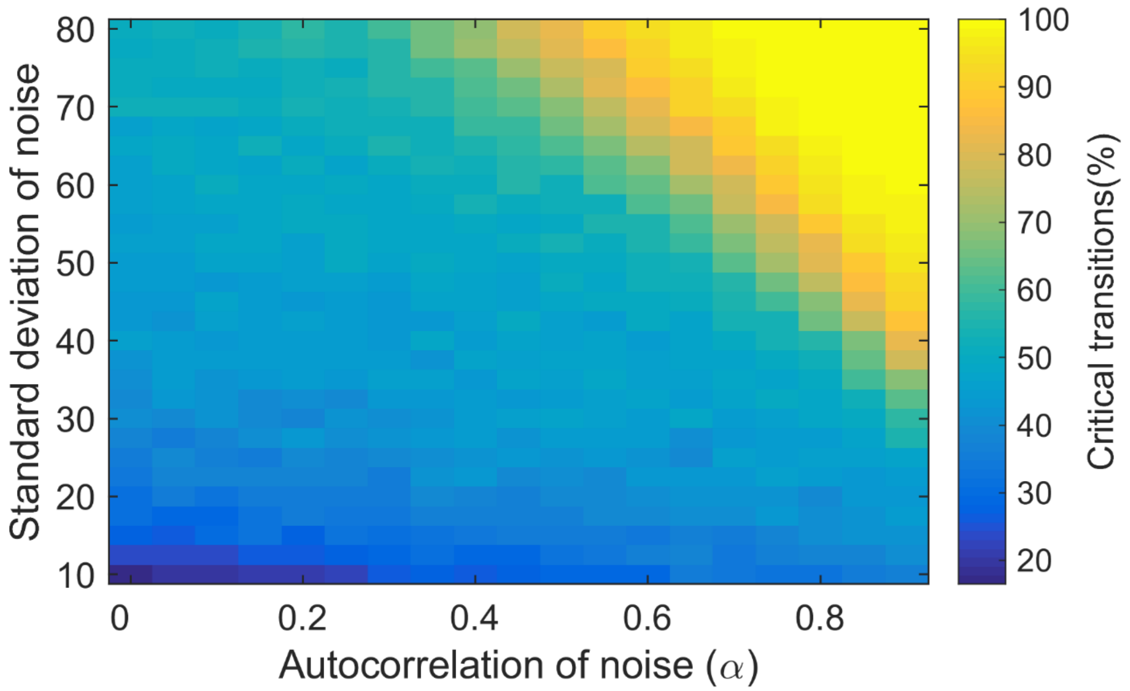

Figure S8. The response of the phytoplankton biomass to an increase in the light intensity with stochastic fluctuations on the light intensity in the model with unsteady drift (scenario 3). Combined effects of autocorrelation and the standard deviation of the fluctuations on the percentage of runs in which the phytoplankton biomass collapses when the rate of change is 0.13 day^{-1}.

S4. Pulse in the incoming light intensity

In the main text the pulse in the incoming light intensity is modelled as a exponential increase to the maximum value, where it will remain for a specific duration, after which it will decrease exponentially to the initial conditions. Because the increase in light intensity is the same as in the unsteady drift scenario (see S2 and Fig. 3), simulations with the same rates (r=0.1 and r=0.17) result in the same trajectories in the phase plane (see Fig. S9). The difference is that because the change in incoming light intensity is temporary, the system will return to the initial biomass, as is visible in Figure S9.

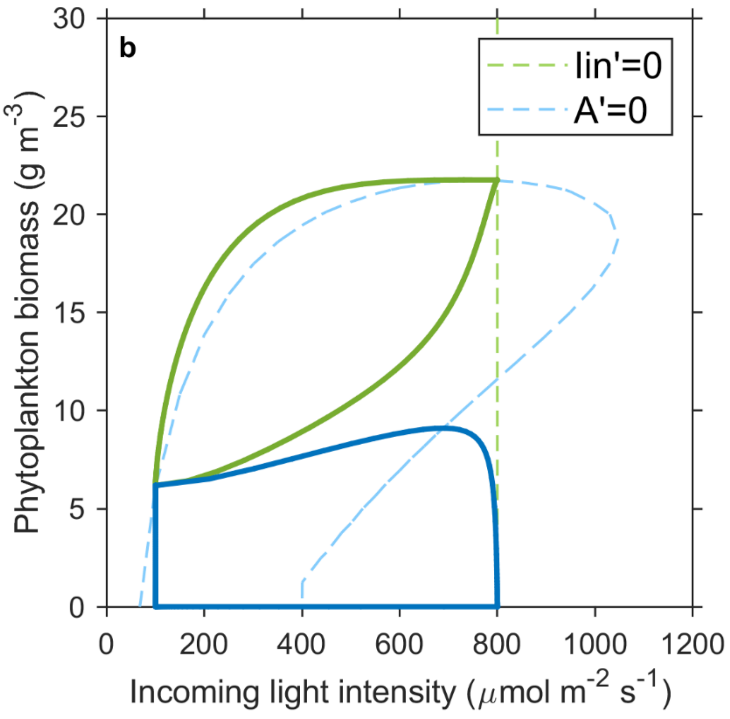

Figure S9. The (I_{in}, A)-phase plane of the model. The blue dotted line is the nullcline of the phytoplankton biomass. The green dotted line is the nullcline for I_{in}, which is a vertical line at $I_{in,max}$ ($I_{in,max}$=800 for these settings). The settings are similar to Figure 3 of the main text (r=0.1 green line, r=0.17 blue line), but instead of exponential increase to $I_{in,max,}$ the change in I_{in} is modelled as a pulse with an exponential increase and decrease after a certain duration.

S5. The effect of scaled environmental variability

In reality, when the time-correlation structure of environmental variability increases, the variance of the variability also changes (see Fig. S10). In our equation for environmental variability without scaling, a decrease in *γ* results in an increase in the variance as a response to weakening feedbacks (reduced *γ*, while *β* stays constant). In the main text we scaled the variance, but as can be seen in Figure S11, the probability of a critical transition increases as the variance of the environmental variability increases. The same holds for an increase in temporal autocorrelation, so increased time-correlation in the environmental variability increases the chance of undergoing a critical transition both by the effect of increased variance and increased temporal autocorrelation.

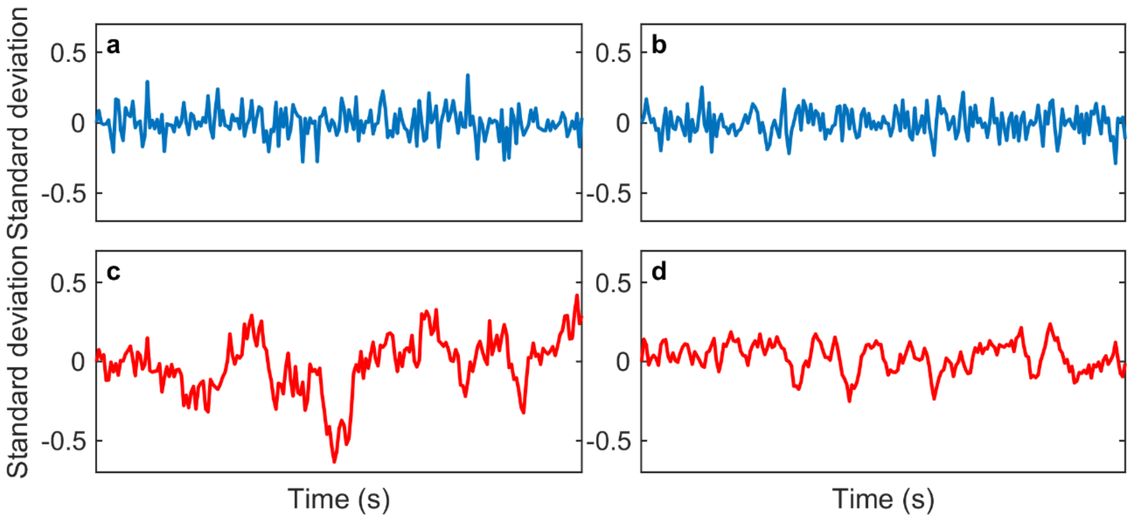

Figure S10. Example runs of environmental variability with and without scaling of the variance. The left-hand panels are without scaling (a, c), and the right-hand with scaling (b, d). The upper panels are time series without time-correlation (blue lines, α=0), and the lower panels (red lines, α=0.8) are time-correlated.

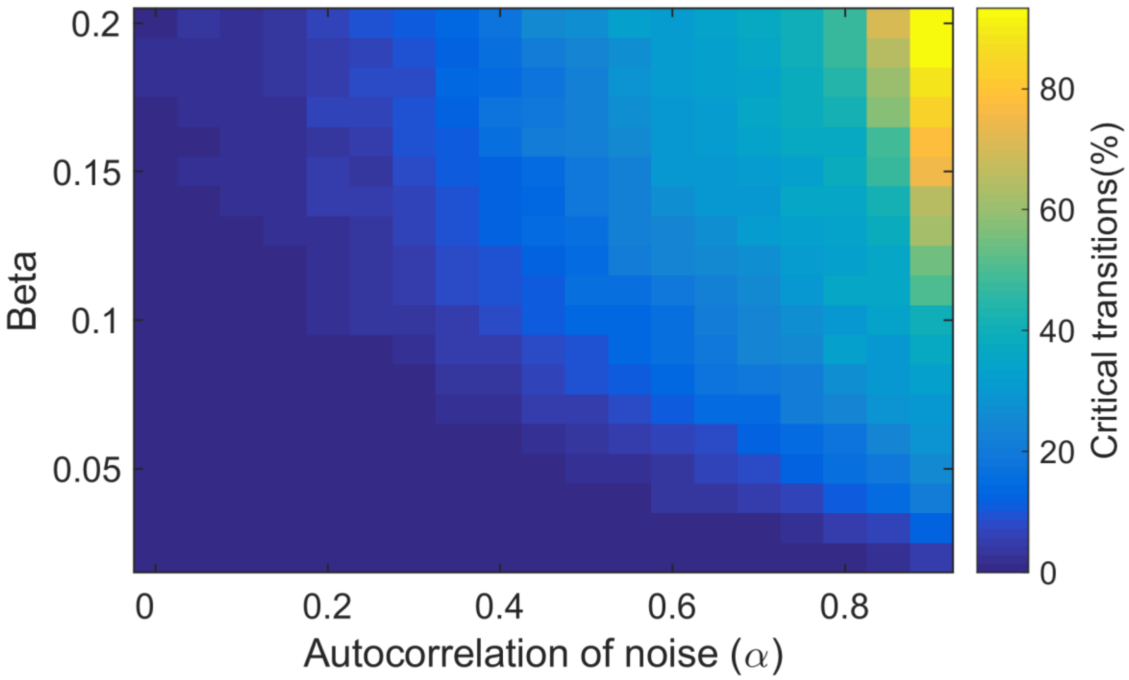

Figure S11. Combined effects of the autocorrelation and the scaling parameter (β) of the fluctuations in the environment on the percentage of runs in which the phytoplankton biomass collapses. The rate of change in the incoming light intensity is 0.12 day^{-1}.

S6. Additional figures

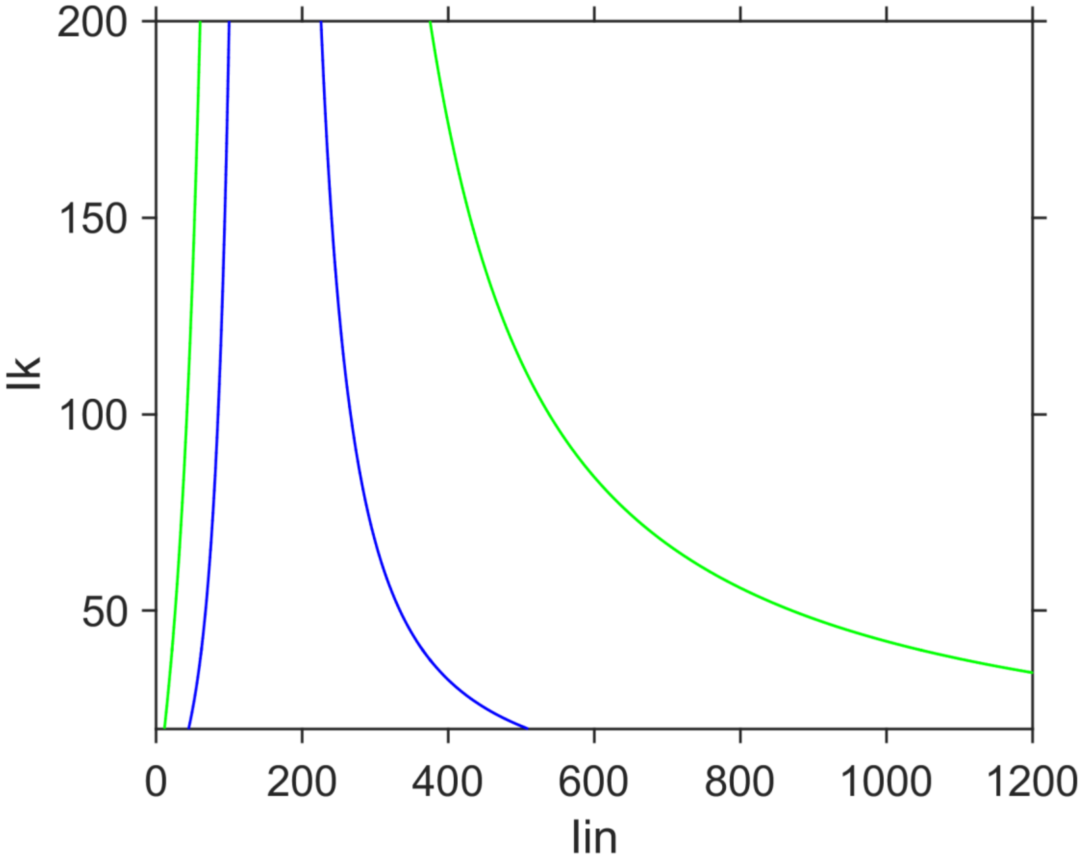

Figure S12. Two-dimensional bifurcation diagram for incoming light intensities between 0 and 1500 µmol m^{-2} s^{-1} and slope of the light intensity curve at Iz=0, between 20 and 200 µmol m^{-2} s^{-1}. The green line shows the fold bifurcations, and the blue line the transcritical bifurcations.

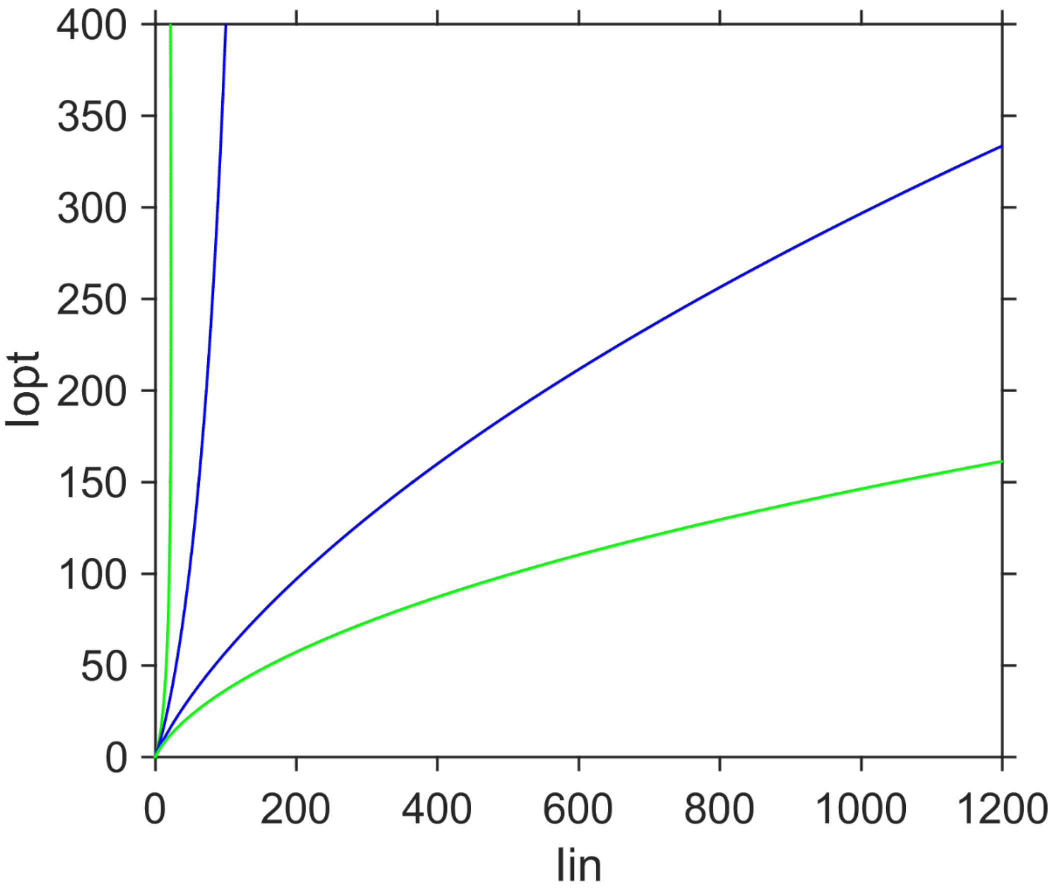

Figure S13. Two-dimensional bifurcation diagram for incoming light intensities between 0 and 1500 µmol m^{-2} s^{-1} and the optimum light intensity of plankton, between 0 and 400 µmol m^{-2} s^{-1}. The green line shows the fold bifurcations, and the blue line the transcritical bifurcations.

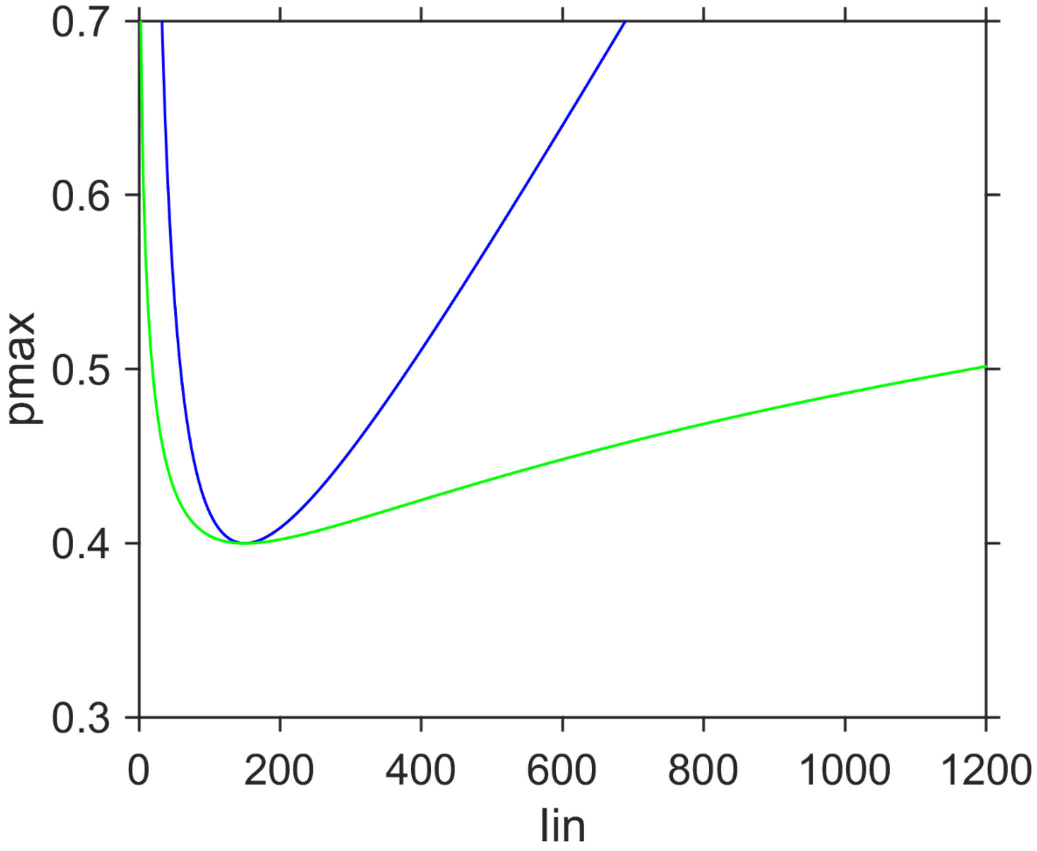

Figure S14. Two-dimensional bifurcation diagram for incoming light intensities between 0 and 1500 µmol m^{-2} s^{-1} and the maximum productivity between 0.3 and 0.7 day^{-1}. The green line shows the fold bifurcations, and the blue line the transcritical bifurcations.
